# Comprehensive user requirements engineering methodology for secure and interoperable health data exchange

**DOI:** 10.1186/s12911-018-0664-0

**Published:** 2018-10-16

**Authors:** Pantelis Natsiavas, Janne Rasmussen, Maja Voss-Knude, Κostas Votis, Luigi Coppolino, Paolo Campegiani, Isaac Cano, David Marí, Giuliana Faiella, Fabrizio Clemente, Marco Nalin, Evangelos Grivas, Oana Stan, Erol Gelenbe, Jos Dumortier, Jan Petersen, Dimitrios Tzovaras, Luigi Romano, Ioannis Komnios, Vassilis Koutkias

**Affiliations:** 1 Institute of Applied Biosciences, Centre for Research & Technology Hellas, Thermi, Thessaloniki Greece; 2 MedCom, Odense, Denmark; 3 Sundhed.dk, Copenhagen, Denmark; 4 Information Technologies Institute, Centre for Research & Technology Hellas, Thermi, Thessaloniki Greece; 5 Department of Engineering, University of Naples “Parthenope”, Naples, Italy; 6 Bit4id S.r.l, Naples, Italy; 7 IDIBAPS, Hospital Clinic de Barcelona, Universitat de Barcelona, Barcelona, Spain; 8 eHealth R&D Unit, EURECAT, Barcelona, Spain; 9 Fondazione Santobono Pausilipon, Naples, Italy; 10 Telbios S.r.l, Milan, Italy; 11 Eulambia Advanced Technologies Ltd, Athens, Greece; 12 CEA, LIST, Point Courrier 172, 91191 Gif-sur-Yvette Cedex, France; 13 Department of Electrical and Electronic Engineering, Imperial College of Science, Technology and Medicine, London, UK; 14 Time.lex, Brussels, Belgium; 15 Exus Software Ltd, London, UK

**Keywords:** Cybersecurity, Interoperability, Health information technologies (HIT), Digital health, Cross-border health data exchange, User requirements engineering, Gap analysis, Barriers and facilitators for HIT acceptance

## Abstract

**Background:**

Increased digitalization of healthcare comes along with the cost of cybercrime proliferation. This results to patients’ and healthcare providers' skepticism to adopt Health Information Technologies (HIT). In Europe, this shortcoming hampers efficient cross-border health data exchange, which requires a holistic, secure and interoperable framework. This study aimed to provide the foundations for designing a secure and interoperable toolkit for cross-border health data exchange within the European Union (EU), conducted in the scope of the KONFIDO project. Particularly, we present our user requirements engineering methodology and the obtained results, driving the technical design of the KONFIDO toolkit.

**Methods:**

Our methodology relied on four pillars: (a) a gap analysis study, reviewing a range of relevant projects/initiatives, technologies as well as cybersecurity strategies for HIT interoperability and cybersecurity; (b) the definition of user scenarios with major focus on cross-border health data exchange in the three pilot countries of the project; (c) a user requirements elicitation phase containing a threat analysis of the business processes entailed in the user scenarios, and (d) surveying and discussing with key stakeholders, aiming to validate the obtained outcomes and identify barriers and facilitators for HIT adoption linked with cybersecurity and interoperability.

**Results:**

According to the gap analysis outcomes, full adherence with information security standards is currently not universally met. Sustainability plans shall be defined for adapting existing/evolving frameworks to the state-of-the-art. Overall, lack of integration in a holistic security approach was clearly identified. For each user scenario, we concluded with a comprehensive workflow, highlighting challenges and open issues for their application in our pilot sites. The threat analysis resulted in a set of 30 user goals in total, documented in detail. Finally, indicative barriers of HIT acceptance include lack of awareness regarding HIT risks and legislations, lack of a security-oriented culture and management commitment, as well as usability constraints, while important facilitators concern the adoption of standards and current efforts for a common EU legislation framework.

**Conclusions:**

Our study provides important insights to address secure and interoperable health data exchange, while our methodological framework constitutes a paradigm for investigating diverse cybersecurity-related risks in the health sector.

**Electronic supplementary material:**

The online version of this article (10.1186/s12911-018-0664-0) contains supplementary material, which is available to authorized users.

## Background

Advances in Health Information Technologies (HIT) and digital health are transforming healthcare delivery. However, the constantly increasing digitalization and the inherent use of sensitive health data come along with the cost of cybercrime proliferation. Lack of adequate security measures result in patients’ and healthcare providers' (HCPs) unwillingness to adopt HIT, as well as investors’ skepticism to fund such activities. In the European context, as the number of citizens who travel across Europe for education, training, work and tourism constantly increases, the need for cross-border health data exchange becomes imperative. Especially, people suffering from chronic diseases are facing obstacles in travelling either within or outside their country of residence, due to the lack of an established, systematic and secure framework for data exchange among healthcare organizations across Europe.

KONFIDO is a European Union (EU) funded project [1], which aims to leverage novel approaches and cutting-edge technologies, such as homomorphic encryption [2], photonic Physical Unclonable Functions (p-PUF) [3], a Security Information and Event Management (SIEM) system [4], and blockchain-based auditing [5], in order to develop a holistic paradigm for secure, cross-border exchange, storage and overall handling of health data. It builds its solution upon existing/evolving European frameworks, such as OpenNCP (Open-source and reference version of the NCP software) [6], which is the open-source National Contact Point (NCP) software implementation of its predecessor project named epSOS (European Partners – Smart Open Services) [7], and eIDAS (electronic IDentification, Authentication and trust Services) [8], which stands for the EU regulation on electronic identification and trust services for electronic transactions in the internal market. An overview of the KONFIDO technical solution and its links with the abovementioned frameworks is presented in [9]. Overall, KONFIDO aims to advance the state-of-the-art of HIT along the four key dimensions of digital security, i.e. data preservation, data access and modification, data exchange, and interoperability and compliance. To this end, KONFIDO is organized in four complementary phases, namely, ‘User requirements analysis; ‘Design’; ‘Technology development’; and ‘Integration, testing and validation’. The current study focuses on the former phase.

As part of the “User requirements analysis” phase, we first reviewed and mapped applicable technical and legal frameworks as well as ethical and social norms at the European level with a major focus on the KONFIDO pilot-site countries (i.e. Denmark, Italy and Spain). This entailed a gap analysis study for interoperable and secure solutions at the systemic level. We then defined and analyzed user scenarios with major emphasis on cross-border health data exchange and, based on these, we conducted a user requirements elicitation phase starting from the definition of the underlying business processes and proceeding to the identification of respective assets, threats and, ultimately, high-level user goals. Equally important, we pursued intense interaction with the wider healthcare community, in order to validate the methods and the outcomes of our approach, aiming also to identify key barriers and facilitators for HIT solutions acceptance linked with cybersecurity. Overall, HIT acceptance in the clinical environment has been identified as a challenge and has been investigated (mostly focusing on Electronic Health Record (EHR) systems [10, 11]), using models based on psychology, sociology, and consumer behavior. To this end, we conducted a survey targeting all possible relevant stakeholders (i.e. HCPs, hospital staff at IT departments, industrial HIT stakeholders, and patients/citizens), as well as an end-user Workshop.

In this paper, we present the overall methodology concerning the user requirements engineering phase of KONFIDO as well as the obtained outcomes. We conclude by consolidating these outcomes in terms of recommendations for the KONFIDO technical design and we argue about the usefulness of the proposed methodological framework for developing secure and interoperable health data exchange IT solutions.

## Methods

**Fig. 1:**
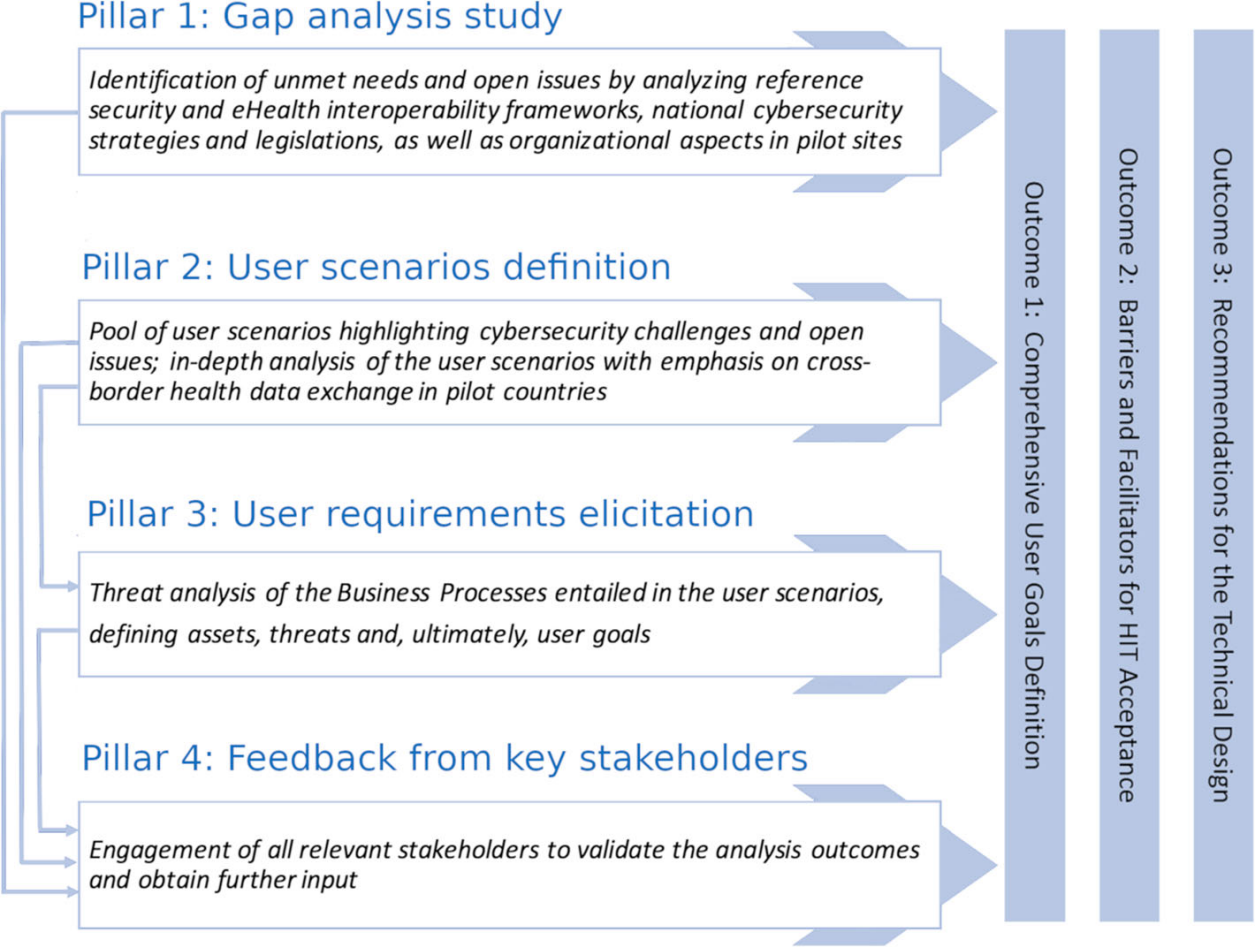
The user requirements engineering framework: methodological pillars and main outcomes

The overall methodological framework adopted for user requirements engineering focuses on four pillars (Fig. 1). The methodological pillars are provided in the left-side of Fig. 1, along with the targeted outcomes in the right-side, while the arrows linking pillars illustrate their interrelations. A description of each methodological pillar is provided in the respective subsections below.

### Pillar 1: Gap analysis study

Generally, a gap analysis aims to identify “gaps”, i.e. the qualitative or quantitative differences, between the *current* and the *target* state of the analyzed subject (e.g. product, process, organization, market, etc.). *Current state* corresponds to the analysis subject’s present status (i.e. “where we are”) and *target state* defines the desired condition where the analysis subject would satisfy some specific criteria or goals (i.e. “where we want to be”). Such an analysis typically requires the comparison of *current* and *target* state across a range of criteria. For the current study, the conducted gap analysis aimed to identify how well our analysis subjects satisfy a set of requirements regarding HIT cybersecurity and interoperability.

The gap analysis subjects included several relevant European initiatives, projects and their outcomes, technological artifacts as well as end-user perspectives and policy strategies across four thematic areas, i.e.:

- *HIT Interoperability Frameworks*: epSOS [7], Antilope [12], the Joint Action to Support the eHealth Network (JASeHN) [13] and SemanticHealthNet [14].
- *HIT Security Software Frameworks*: DECIPHER [15], OpenNCP and STORK 2.0 [16].
- *End-user perspectives across diverse settings in KONFIDO pilot countries*: Santobono Pausilipon Hospital (Italy), Odense University Hospital & Svendborg Hospital (Denmark), and Hospital Clínic Barcelona (Spain).
- *National cybersecurity strategies and reference reports*: Documents regarding the currently applied cybersecurity strategies in the pilot countries and relevant reports (e.g. regarding guidelines or best practices) primarily provided by the European Union Agency for Network and Information Security (ENISA) [17, 18].

The analysis was assigned to Working Groups (WG) per thematic area within the KONFIDO Consortium. The analysis subjects were examined by topic experts in each WG against a gap analysis template (provided in Additional file 1). This gap analysis template defined an explicit set of analysis criteria (a.k.a. *controls*), mostly based on the ISO/IEC 27k information security standards family [19]. In the scope of the presented study, the following ISO standards were employed: (a) ISO/IEC 27002 [20]; (b) ISO/IEC 27010 [21]; (c) ISO/IEC 27040 [22]; (d) ISO 27799 [23]; (e) ISO 22857 [24], and (f) ISO/IEC 25010 [25].

The template was organized on 11 clauses, defining the template’s upper–level structure: *Security policy; Organizing information security; Asset management; Human resources security; Physical and environmental security; Communications and operations management; Access Control; Information systems acquisition, development and maintenance; Information security incident management; Business continuity management; Compliance,* and *Usability.* Instructions and relevant examples on how to use the template were given to the WGs. In addition, the respondents became aware that some questions contained in the template might not be relevant for their analysis, due to the specific scope and/or the varying information granularity of the considered analysis subjects. Finally, iterative teleconferences were conducted among WG members to discuss the plan, their progress, and finalize the results.

The gap analysis was mainly conducted via: (a) *Desk research*, by reviewing material regarding the analysis subject, e.g. project reports or deliverables, as well as papers published in scientific journals or conferences. (b) *Interviews / discussions with experts* related with the analysis subject (either directly involved in KONFIDO or not). The overall gap analysis methodology along with some preliminary results were presented in [26].

### Pillar 2: User scenarios definition

Given the European dimension of KONFIDO, its user scenarios focus on cross-border health data exchange. In particular, two reference scenarios have been defined, the first focusing on cross-border services for a chronic patient, and the second elaborating on cross-border and cross-regional health data exchange, considering triage services in emergency situations. Several stakeholders have been taken into account in the scenarios’ definition, e.g. public and private hospitals, HCPs with different roles and patients with diverse healthcare needs, as well as various technological artifacts (mHealth apps, telemonitoring services, EHRs, etc.). The aim was to address the heterogeneity of the domain, considering the three pilot countries of the project. The second scenario is described in Table 1 [27].

**Table 1:** The second reference scenario considered in KONFIDO

Phase 1: Milan Anna is a 45-year-old university professor living in Milan (Lombardy Region), Italy. For the summer holidays, Anna and her daughter are planning a cruise to Barcelona, Spain. Anna suffers from Diabetes type 2, while her 6-year-old daughter Cristina has heart disease since she was born. Being a chronic patient, Anna has learnt how to live with her disease and to manage her daughter’s health too, undertaking routine tasks such as **measuring** periodically Cristina’s vital signs (e.g., blood pressure), **taking** medicines, or **performing** tasks like glucose measurements and insulin injections. Cristina was enrolled in the Regional Program called CReG (Chronic Related Groups) and together with her mother they use a tele-monitoring service. CReG is a program which delegates the care management of chronic patients to General Practitioners, supporting them in the prescription, monitoring and renewal of care plans. The hospital of Milan has equipped both Anna and Cristina with a tele-monitoring kit for **remote monitoring** of their health condition. The kit includes medical devices and a gateway which sends the measured vital signs to the respective Service Center in Milan. Phase 2: Naples Travelling by car for a conference in Naples (Campania Region) with her husband and their daughter, Anna experiences a quite serious car accident and Cristina has serious wounds. The healthcare authorities in Naples, where the accident takes place, offer an innovative telemedicine application empowered by KONFIDO. Particularly, using the national eID technology that KONFIDO recognizes and handles properly, the retrieval of all the information needed to intervene while in the ambulance (patient identification, clinical details, immunization details, and usual therapy) is made possible. Specifically, Cristina’s data are retrieved from the EHR system of the healthcare authorities in the Lombardy Region. Using the telemedicine application and a tablet, Cristina’s personal data (including pictures of her wounds) are **transmitted** through the mobile network to the emergency department by paramedics. Using KONFIDO technologies, paramedics can safely **authenticate** her and the encrypted transmission of her medical data is conducted. The application **monitors** the child, **suggests** actions, possibly re-routes the ambulance, and makes sure that everything is ready upon arrival at the hospital with the aim to speed-up the triage process and reinforce the preparedness levels. Phase 3: Barcelona After a few weeks, Cristina is discharged from the hospital in Naples and, given her risky heart condition, the doctor in Milan **is immediately informed** by the hospital in Naples that anti-coagulant therapy had to be interrupted. Consequently, the doctor decides to adjust the therapy and **review the monitoring plan**. Cristina and Anna can realize their vacation plans in Spain using the tele-monitoring service. Anna and Cristina know that in case of problems, any hospital they might have to visit in Barcelona will **have access** to their patient summaries in Italy. During the journey, Anna faints and she is transferred to the nearest hospital in Barcelona to check her health condition. While the Spanish doctor is accessing Anna’s patient summary, a cyberattack tries to compromise the data exchange. Specifically, an international hacker group, using a system vulnerability, attacks and takes control of the NCP in the Spanish OpenNCP deployment. Thanks to KONFIDO security mechanisms, Anna’s data integrity and confidentiality is protected against the cyberattack and the doctor can make a diagnosis and provide the medical treatment.	

Terms highlighted in bold indicate verbs or phrases that have been used to identify the respective BPs in the scenario

Besides the textual description of each user scenario, a workflow was defined (for its realization) and analyzed in detail.

### Pillar 3: User requirements elicitation

The term “user requirements elicitation” can be ambiguous in the varying contexts of user requirements engineering. In the scope of this work, we defined it as the process of exploiting diverse information sources, in order to “… *discover the current project needs and agree upon its vision and goals*” [28]. Our overall approach aimed at specifying high-level *user goals* by first defining the related *business processes* (BPs), based on the methodology described in Park et al. [29]. User goals in turn are defined as “*abstract user requirements, not directly referring to specific technical solutions or components*”. They typically refer to specific user actors, while their definition facilitates early identification of possible conflicts between actors and, consequently, their timely resolution.

The identification of BPs was based on the actions contained in the textual description of each scenario, which is a well-established approach [30]. Typically, “*verbs correlate to operations which can be invoked by components or actors*” [31], in order to facilitate the specification of the system functionality. The user scenario presented in Table 1 is annotated based on the above rationale by highlighting in bold the key-phrases implying BPs. The identified BPs were then analyzed by conducting a *threat analysis*. Typically, this refers to the systematic process of identifying and evaluating spots of vulnerability for a facility, operation, or system, which is also applicable in the context of HIT [32, 33]. Our threat analysis process involved the following steps:

1. *Asset identification*; assets include anything worth to be protected and can be organized in the following indicative categories: *information*, *infrastructure* (physical infrastructure, software, etc.), *persons*, *business functions*.
2. *Threats identification*; threats are uncontrolled circumstances or actions, typically related with malicious people or factors out of control (e.g. weather, physical failures, etc.), which can obtain control of, damage or destroy an asset.

Threats may refer to technical, functional, legal, personal and political aspects. We focused on technical threats, which were classified based on the STRIDE model [34]:

- ***S****poofing*: refers to gaining access to a system by using a false identity.
- ***T****ampering*: refers to the unauthorized modification of data.
- ***R****epudiation*: refers to the denial of specific actions or transactions on the user’s behalf (legitimate or not).
- ***I****nformation disclosure*: refers to exposure of private or sensitive data.
- ***D****enial of service (DoS)*: refers to the process of making a system/application unavailable.
- ***E****levation of privilege*: refers to gaining access to resources by self-assigning more privileges.

The threat analysis results were combined with best practices and outcomes produced by relevant projects/initiatives, in order to define the user goals per actor. In particular, we took into account: (a) the ISO/IEC 27k family of standards; (b) outcomes of the gap analysis, the end-user survey and Workshop conducted in the scope of the project, as described below; (c) reports from relevant EU projects and initiatives, as well as (d) the recently enforced into practice General Data Protection Regulation (GDPR) [35], which aims to align data privacy laws among EU Member States.

We defined two types of user goals, i.e. *functional* and *non-functional*, corresponding to functional and non-functional requirements, respectively. Functional goals were based on the user scenarios, while the identified threats per BP were combined with other sources of information to pinpoint non-functional goals.

Aiming to consolidate and interpret the identified user goals, a meta-analysis was conducted based on a visual analytics approach. The aim was to illustrate the dependencies among the identified user goals and the respective information sources, BPs, assets and threats, as well as the strongest links among them.

### Pillar 4: Feedback from key stakeholders

An end-user engagement strategy was employed to validate the prior methodological pillars and their outcomes, and identify key barriers and facilitators for HIT adoption linked with security and interoperability. In particular, an online, anonymous and confidential survey as well as a Workshop with the participation of key stakeholders were conducted. The goal of the survey was two-fold: (a) to identify the currently applied practices regarding security and interoperability on existing HIT infrastructures for healthcare organizations of varying size and nature (e.g. private and public), and (b) to obtain insights regarding patient/citizen awareness on cybersecurity risks entailed in cross-border health data exchange and document opinions about exchanging health data with HCPs or HIT service providers. Thus, we discriminated two groups of participants in the survey: (a) *HCPs and HIT stakeholders across Europe*, and (b) *patients/citizens*.

The overall survey design was built upon key principles of human psychology [36], while it contained different content per group. Several sources were used for designing the respective questionnaires, such as relevant standards, surveys conducted by other organizations, reports, scientific papers, etc. The questionnaire structures for both participant groups are provided in Additional files 2 and 3, respectively.

For the first group, personal invitations were sent, in order to obtain high-quality, expert feedback. The survey questions for this group were structured as follows:

1. *Organization profile*: referred to the organization’s size and structure (e.g. number of employees, activities in the domain, etc.).
2. *Security facts*: focused on security incidents occurred in the organization, targeting IT stuff and managers.
3. *Security policy*: referred to policies applied in the organization (e.g. existence of security and risk management policies, use of encryption, etc.).
4. *Security incident management*: concerned handling security breaches in a technical level, targeting technical stuff and managers.
5. *Barriers and facilitators*: aimed to identify key issues that facilitate or discourage the adoption of cybersecurity best practices.
6. *Personal view*: focused on awareness (e.g. use of publicly available cloud storage services, importance of security in everyday work, etc.) and satisfaction regarding the current cybersecurity state.

Contrary to the survey targeting the first group, the survey for the second group was circulated publicly by using patient forums, mailing lists, and social media. It contained the following sections:

1. *Awareness regarding Information Technology risks*: Focused on identifying the level of the participants’ awareness regarding the risks entailed in using HIT.
2. *Legislation*: Aimed to identify the patients’/citizens’ familiarity with relevant legislation artifacts.
3. *Cross-border medical treatment*: Aimed to provide insights on whether the participant was medically treated or hospitalized abroad.
4. *Cross-border medical data exchange*: Focused on the participants’ opinion regarding the need for cross-border health data exchange.
5. *Barriers and facilitators*: Aimed to identify issues that facilitate or discourage cross-border health data exchange from a patient’s/citizen’s viewpoint.
6. *Demographics*: Contained key information about the participant, in order to facilitate the statistical analysis of the obtained data.

The end-user Workshop attracted more than 30 stakeholders from the HIT and healthcare sectors across Europe; it was organized to encourage open discussion, exploring the diverse issues concerning cross-border health data exchange. Personal invitations were sent to candidate participants from diverse organizations (healthcare, standards developing organizations, HIT associations, regional healthcare authorities, privacy authorities, research/academia, etc.), aiming to obtain input from the widest possible spectrum of stakeholders composing the European HIT ecosystem. The methodological overview along with preliminary outcomes as regards barriers and facilitators for HIT acceptance were presented in [37].

## Results

In this section, we present the main outcomes of the employed methodology (Fig. 1). Given the wide range of the activities carried-out in the scope of this work, we concentrate on the key parts of the findings.

### Outcome 1: Comprehensive user requirements definition

**Fig. 2:**
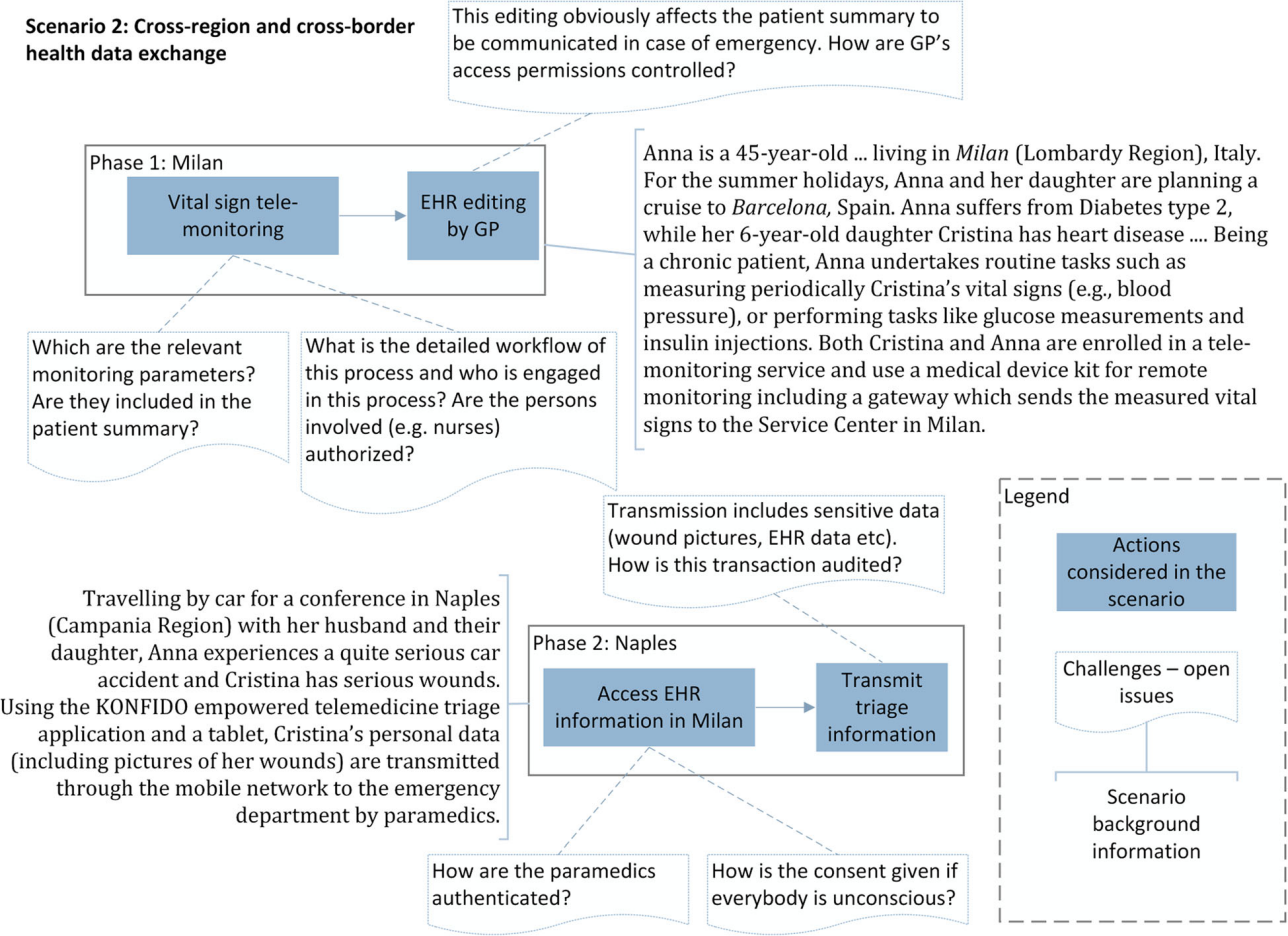
Partial view of the workflow corresponding to the user scenario described in Table 1

The detailed analysis of the user scenarios highlighted the challenges and the open issues of applying them in real-world settings, taking into account the context of the project’s pilot sites. Figure 2 depicts a part of the workflow corresponding to the user scenario presented in Table 1, highlighting actions considered in the senario, the entailed challenges and open issues, as well as scenario background information.

The user scenarios analysis resulted in a set of BPs (listed in Table 2). In order to illustrate the user goals definition process, we present the analysis of BP2: “Access the medical record of a foreign patient”, demonstrating this way indicative results in each step of the analysis (Table 3 depicts the assets and Table 4 the identified threats for BP2, respectively).

**Table 2:** Business processes identified from the user scenarios

ID	Business process	Description
BP1	Grant access to own Medical Record	A patient in the visiting country grants the foreign HCP access to his/her medical record to facilitate treatment.
BP2	Access the medical record of a foreign patient	The HCP accesses a foreign patient’s medical record, e.g. his/her medical history summary, medication treatment plan, diagnosis and relevant lab examination results.
BP3	User authentication using the national eID infrastructure	The user is being authenticated via his/her nationally-issued eID.
BP4	Transmitting data for remote monitoring	The user transmits data using a telemonitoring service.
BP5	Accessing the patient’s medical record while transferred via an ambulance	Paramedics retrieve data from the patient’s medical record.
BP6	Exchanging triage information, while the patient is transferred to the hospital via an ambulance	Paramedics transmit triage data to the respective hospital, e.g. wound pictures. The application transmits patient data in the ambulance and may provide guidance to the paramedics.
BP7	Exchange of medical information between HCPs	HCPs exchange medical information directly, e.g. in the case of a medication safety issue, and notify the treating physician accordingly. This BP refers to an active way of communication and not to keeping notes in the patient’s medical record.

**Table 3:** Assets identified for BP2: “Access the medical record of a foreign patient”

ID	Description	Category	Comments
A1	Medical record information	Information	The main asset to be protected.
A2	HCP credentials	Information	e.g. usernames, passwords etc.
A3	HCP authentication means	Infrastructure	e.g. eID card
A4	Intention of accessing medical record	Information	The intention of accessing a patient’s medical record is crucial. On the one hand, it could imply an attack attempt and, in this case, the medical record owner should be notified. On the other hand, it should be protected as it clearly implies that the doctor intends to conduct a medical transaction, and this could contain sensitive information.

**Table 4:** Threats identified for BP2: “Access the medical record of a foreign patient”

ID	Type	Assets	Malicious actors	Description/Example scenario
T1	Spoofing	All information assets	Other actors without a clear role in the BP	An external actor could pretend to be legitimate, in order to get the HCP credentials and use them to access information (e.g. patient’s medical record), on behalf of the HCP.
T2	Tampering	All information assets	Other actors without a clear role in the BP	A malicious user could (perhaps combined with a spoofing attack) modify the information assets (e.g. the patient’s medical record or the HCP’s credentials) in a malicious way for social, financial or for personal reasons.
T3	Repudiation	All information assets	HCPs	Deny accessing medical information to avoid legal consequences upon an HCP (e.g. in a case of a medical error).
T4	Information disclosure	All information assets	HCPs and other actors without a clear role in the BP	An HCP could provide access to a patient’s medical record, aiming at patient’s financial or personal harm or for personal financial benefit.
T5	Denial of Service	Medical record information	Other actors without a clear role in the BP	Hinders access to the respective services, aiming to cause damage to the patient or the healthcare organization providing the medical services.
T6	Privilege Elevation	Medical record information	Other actors without a clear role in the BP	Assign privileges to one or multiple medical records aiming at exploiting or damaging data, or alternatively aiming at patients’ financial or personal harm.
T7	Physical stealing	Physical authentication means	Other actors without a clear role in the BP	Stealing the eID card of the HCP could facilitate spoofing, information disclosure and privilege elevation.

These threats were analyzed, taking also into account further information sources, e.g. ISO standards, guidelines produced by other European projects, etc. Based on our analysis, a set of 30 user goals were defined in total (Tables 5 and 6 demonstrate two example goals associated with BP2).

**Table 5:** Goal 11: “Prevent tampering attacks”

G11	Prevent tampering attacks
Goal Type	Non-functional
Actor(s)	Other actors without a clear role in the BP
Reference(s)	BP2
Description	As someone could (perhaps combined with a spoofing attack) modify the information assets (e.g. the patient’s medical record or the HCP’s credentials) in a malicious way for social, financial or personal reasons, KONFIDO should be able to prevent such kind of malicious actions.

**Table 6:** Goal 12: “Prevent ambiguity issues”

G12	Prevent ambiguity issues
Goal Type	Functional
Actor(s)	HCP, Patient
Reference(s)	BP1, BP2, JASeHN deliverable D5.3 (section IV)
Description	Semantic ambiguity can be a burden in cross-border health data exchange. Referencing to diseases and medication might be confusing in clinical practice due to different drug brand names, clinical protocols/procedures, etc. KONFIDO should promote semantic interoperability in order to minimize these risks.

**Fig. 3:**
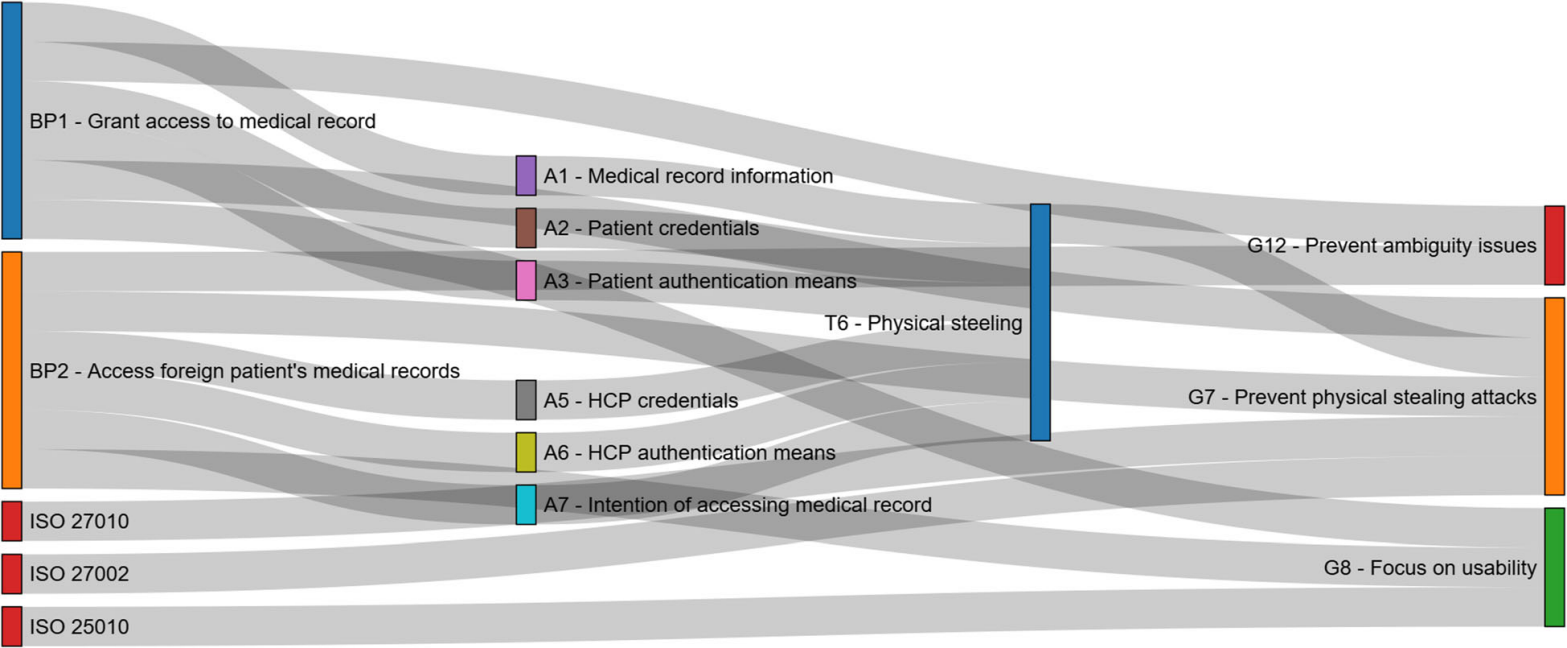
Sankey diagram illustrating an indicative view of links among specific information sources considered in the analysis

**Fig. 4:**
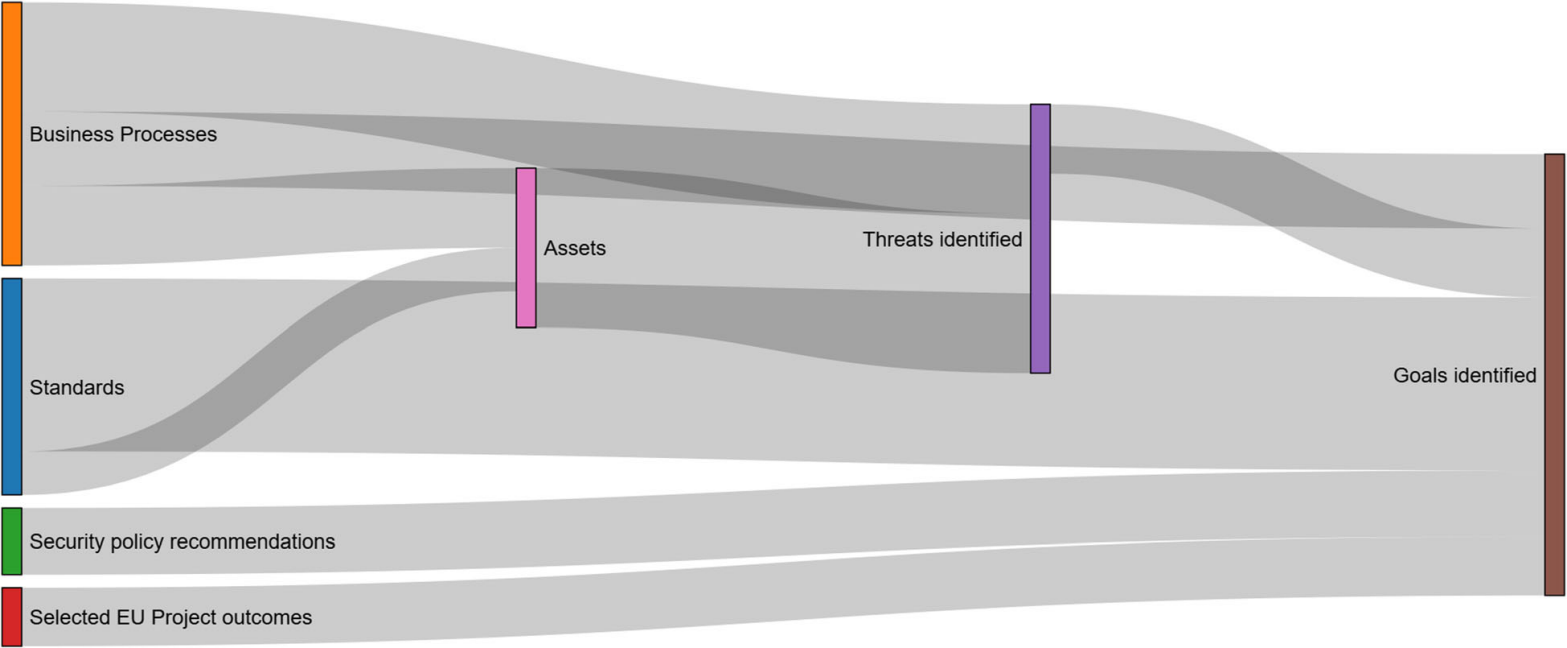
Sankey diagram illustrating the overall contribution of the categories of information sources considered in the analysis for goal definition

Aiming to further analyze the identified user goals, we conducted a meta-analysis using visual analytics. Diagrams demonstrating the link among the outcomes of intermediate analysis steps (i.e. assets and threats), the original information investigated (i.e. standards, policy recommendations, etc.) and the final user goals, were produced. This visualization highlighted the complexity of these links for specific intermediate outcomes, information sources and user goals, and gave a broader overview concerning the overall contribution in the user goals’ definition by grouping the intermediate analysis steps and the original information investigated according to their category. Figure 3 provides an indicative example visualization, depicting a subset of the links among information sources (e.g. standards, BPs, reports on the left side of the figure), intermediate outcomes (assets and threats, in the middle) and user goals G7, G8 and G12 (on the right side of the figure). Respectively, Fig. 4 provides an example visualization depicting the overall contribution of the categories of information sources considered in our analysis (left side of the figure) and the categories of intermediate analysis steps (i.e. assets and threats, in the middle) in the user goals definition (right side of the figure).

Table 7 demonstrates the quantified contribution of the most important information categories. Evidently, ISO standards were the most influential source of information in this respect.

**Table 7:** Origin of user goals with respect to the information asset categories considered in our meta-analysis

Original source category	Percentage of goals referring to the category
Standards	29%
Business Processes	24%
Threats	13%

### Outcome 2: Barriers and facilitators for HIT acceptance

The gap analysis study provided the initial input for this outcome, since it revealed barriers and constraints as well as open issues and challenges for information security in the health sector. This input has been further elaborated in the Workshop and also through the conducted survey. As an example, we present the analysis of the everyday operational processes applied in one of the KONFIDO pilot sites, the Santobono Pausilipon Hospital (PAUSIL) in Naples, Italy. PAUSIL is a specialized pediatric hospital with more than 1000 employees and it demonstrated high adherence to the controls contained in the gap analysis template and the respective underlying standards. Nevertheless, some indicative gaps were identified (Table 8).

**Table 8:** Indicative gaps identified in the PAUSIL hospital

Gap analysis template clause	Gap analysis objective	Question/security control	Current status and gap mitigation
Security Policy	Information security policy	Does the analysis subject facilitate or promote the idea of information security policy document?	A formal information security policy document does not yet exist; however, PAUSIL is planning to introduce operational procedures and policies regarding security.
Physical and environmental security	Secure areas	Does the analysis subject facilitate or promote protecting against external and environmental threats?	Protection against external and environmental threats is not centrally documented/planned.
Usability	Effectiveness	Does the analysis subject facilitate or promote the operability regarding the respective security aspects?	The process of changing user passwords could be improved in terms of usability.
Communications and operations management	Media handling	Does the analysis subject facilitate or promote management of removable media?	No formal procedures are enforced for the management of removable media

Overall, the main issues identified through the gap analysis for the considered analysis subjects can be summarized as follows:

1. Full adherence to the targets set by international standards for information security is currently not universally met. For example, the processes applied in the considered hospitals demonstrated high adherence with the controls proposed by information security standards. However, compliance with standards was not evident in the review of the considered interoperability and software security frameworks.
2. The analysis of national cybersecurity strategies and reference reports highlighted the difficulty in balancing between a high-level document and actionable information. As a consequence, this material can be ambiguous for users and, therefore, the adherence is partly incentivised and arbitrarily localized.
3. As technology evolves at rapid pace, cybersecurity artifacts can quickly become outdated. A sustainability plan for the employed technologies should be undertaken, in order to enhance user trust. In some cases, legacy or vulnerable technologies were identified in the investigated technology frameworks.
4. Lack of integration towards a holistic security approach was clearly identified. While, various interesting technologies are being developed in parallel, it seems that each project focuses on a specific technological aspect and integration is not taken into account to leverage cybersecurity of HIT as a whole.

**Fig. 5:**
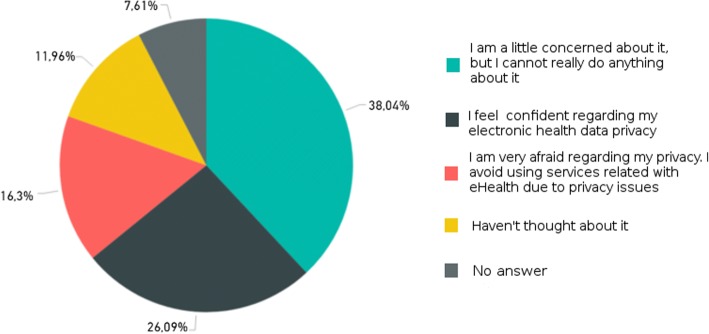
Answers to question “Have you ever thought about your privacy regarding your health data?”

**Fig. 6:**
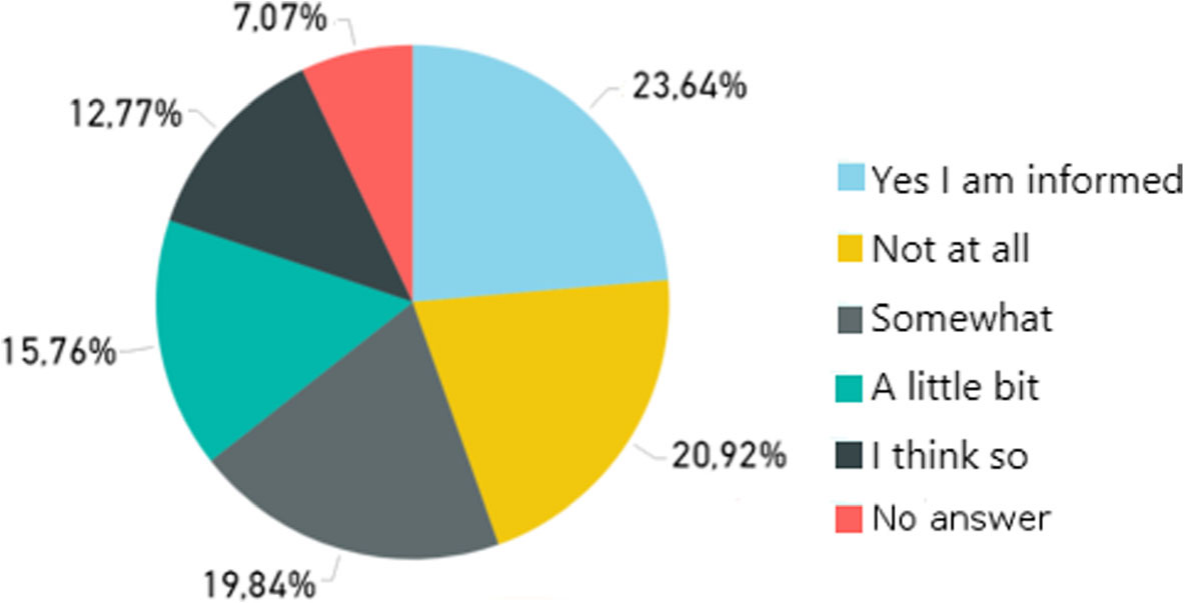
Answers to question “Do you feel well-informed regarding possible health data security risks?”

The survey focusing on selected stakeholders, i.e. HIT experts, managers, HCPs and health IT stuff working in hospitals, resulted in 35 submissions. The open survey targeting patients/citizens attracted 437 submissions. The analysis of the submitted responses led to the identification of barriers and facilitators regarding HIT acceptance. For example, barrier B1: “*Lack of awareness regarding information technology risks”* was identified due to the analysis of the responses to the questions “*Have you ever thought about your privacy regarding your health data?*” (depicted in Fig. 5) and “*Do you feel well-informed regarding possible health data security risks*?” (depicted in Fig. 6), provided by the patients/citizens group.

**Fig. 7:**
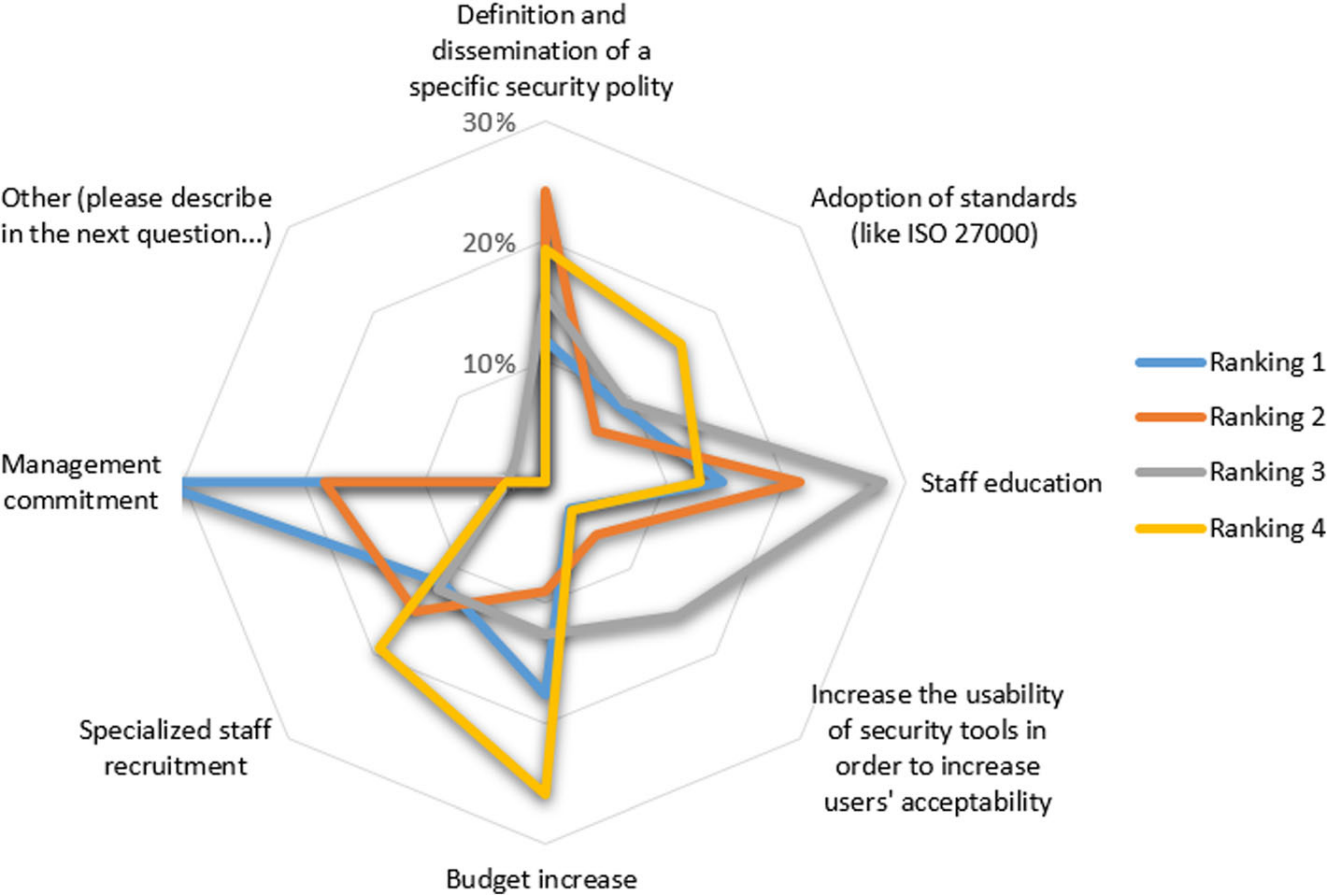
Answers to question “Please rank the importance of the issues that you think might facilitate the adoption of security-oriented best practices” (for readability, only the most popular responses are presented)

**Fig. 8:**
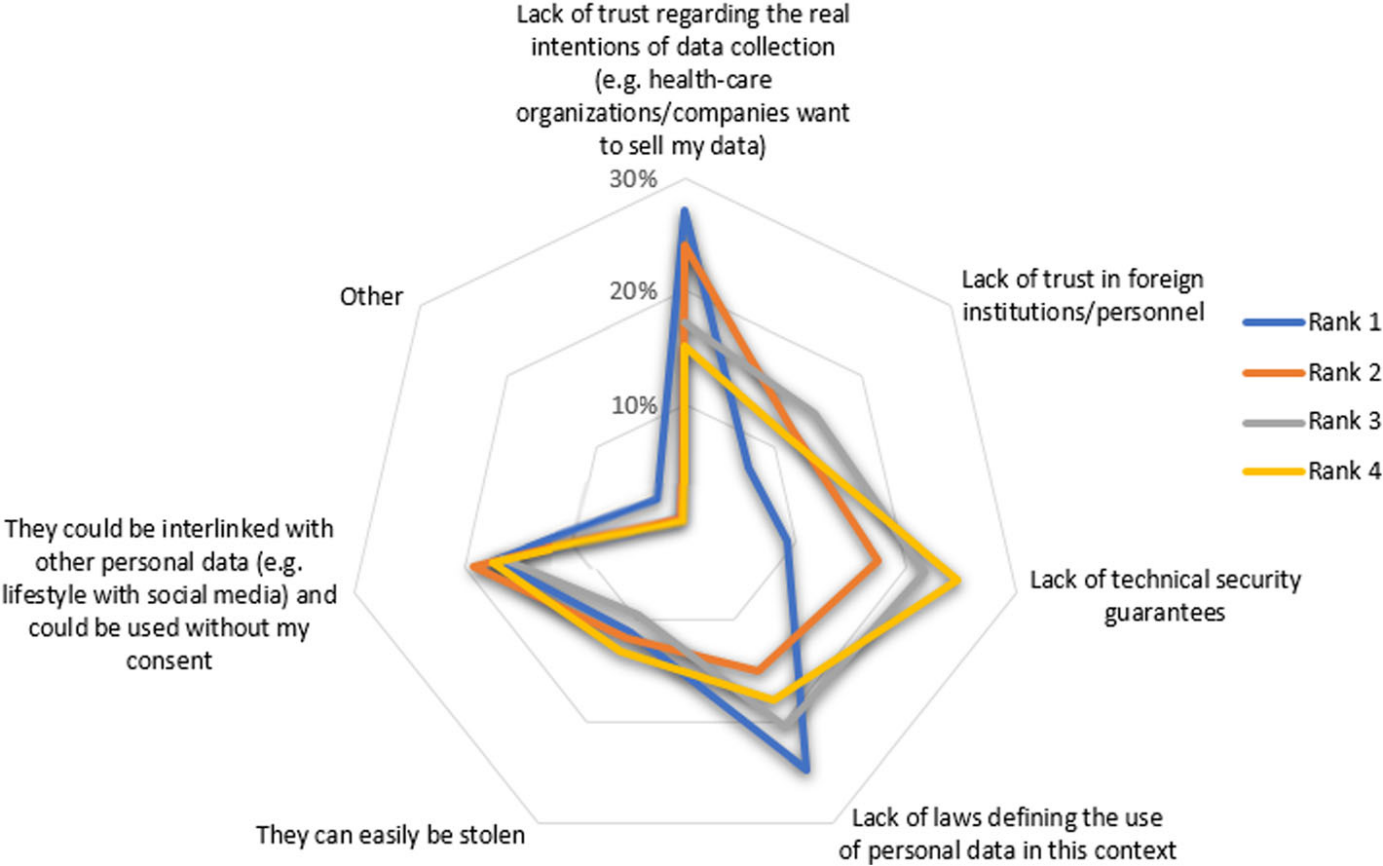
Answers to question “Please rank the following barriers, hindering acceptance of cross-border health data exchange”

As another example, facilitator F6: “*Wide recognition of the need for a security policy based on standards*” was partly identified due to the responses to the question “*Please rank the importance of the issues that you think might facilitate the adoption of security-oriented best practices*” provided by the selected stakeholders group (Fig. 7). Similarly, answers to question “*Please rank the following barriers, hindering acceptance of cross-border health data exchange*” provided by the patients/citizens group (Fig. 8) were linked with barrier B2: “*Lack of end-user confidence on their overall electronic health data handling*”.

Overall, the analysis of the survey responses and the outcomes of the Workshop led to the identification of a comprehensive set of barriers and facilitators regarding HIT acceptance (shown in Tables 9 and 10, respectively). The barriers identified in Table 9 are grouped with respect to awareness, interoperability, legislation, trust, and usability.

**Table 9:** Barriers for HIT acceptance linked with cybersecurity and interoperability

ID	Description	Expected impact on technical design and/or the overall KONFIDO project activities	Category
B1	Lack of awareness regarding information technology risks	Need to reinforce awareness on cybersecurity risks associated with healthcare delivery.	Awareness
B2	Lack of end-user confidence on their overall electronic health data handling	The technical design shall account for a comprehensive and transparent data handling scheme.	Trust
B3	Lack of trust to private companies providing HIT services	The solution should focus on using infrastructure in the most transparent way possible.	Trust
B4	Lack of interest regarding the “Terms and Conditions” for using HIT services	▪ Need to make “Terms and Conditions” more comprehensive for all users. ▪ Need to support the implementation of a comprehensive and transparent data handling scheme.	Trust
B5	Inadequate level of legislation awareness	Need to promote awareness on legislation aspects.	Awareness
B6	Lack of perceived effectiveness of legislation by end-users	Need to explain and illustrate the effectiveness of legislation to end-users.	Trust
B7	Lack of clear and transparent consent processes currently applied	Need to design a comprehensive consent mechanism.	Trust
B8	Legislation not aligned among EU Member States	Need to track ongoing legislation initiatives and adapt the technical design accordingly.	Legislation
B9	Immaturity of existing frameworks	Need to reduce strong dependencies with such frameworks to the extent possible.	Usability
B10	Partial lack of management commitment	Need to raise awareness on cybersecurity risks associated with healthcare delivery.	Awareness
B11	Lack of a cybersecurity-oriented culture in everyday operations	Need to raise awareness on the cybersecurity risks associated with healthcare delivery.	Awareness
B12	Lack of budget	Need to raise awareness on the impact of cybersecurity incidents and the economic burden that these may entail.	Awareness
B13	Usability reduced due to IT security measures	Need to prioritize usability in the technical design process.	Usability
B14	Inadequate use of established cybersecurity mechanisms (e.g. active directory, intrusion detection systems, etc.)	Need to promote the use and added value of novel/standard cybersecurity mechanisms.	Awareness
B15	Diversity of information workflows among organizations	Need to contextualize the technical design, in order to accommodate the requirements of local healthcare delivery processes and therefore increase end-user acceptance through enhanced usability.	Usability
B16	Free-text content in different languages	Need to employ reference medical terminologies/encodings to address interoperability.	Interoperability
B17	Legislation not aligned among EU Member States	Need to follow ongoing legislation initiatives and adapt the design according to EU directives.	Legislation
B18	Legal issues not clarified (e.g. data ownership, liability etc.)	Focus on provenance and auditing mechanisms, in order to clarify details if/when needed and, therefore, increase trust on the overall data exchange process.	Legislation
B19	Lack of inter-organizational trust	Need to promote robust and transparent cybersecurity measures while illustrating the added value of health data sharing (e.g. considering patient safety, quality of care, etc.).	Trust
B20	Complexity of consent process	Need to design a comprehensive consent mechanism for patients.	Usability
B21	Lack of available IT expertise in organizations	Need to raise awareness about the required personnel to address cybersecurity risks in organizations delivering healthcare services.	Awareness
B22	Data exchange agreement’s complexity	Need to establish data exchange agreements compliant with legal norms.	Usability

**Table 10:** Facilitators for HIT acceptance linked with cybersecurity and interoperability

ID	Description	Expected impact on technical design and/or the overall KONFIDO project activities
F1	The need for HIT services and applications tends to overcome the insecurity regarding personal data misuse	It confirms the need for solutions that provide added value in real-world healthcare settings, while still promoting a holistic security approach.
F2	End-users support cross-border data exchange (even for research)	It confirms the value of the KONFIDO key concepts. Does not affect design decisions.
F3	Common legislation activities between EU Member States	GDPR and other initiatives will form the legal base for the solution and guide the respective design decisions (e.g. on the consent process).
F4	Technical EU initiatives are currently ongoing	The design will create a liaison with and build upon existing/evolving frameworks in Europe (epSOS, OpenNCP, eIDAS).
F5	Standards already established and widely accepted	The design and implementation will follow security standards, such as those from ISO/IEC 27k.
F6	Wide recognition of the need for a security policy based on standards	The technical solution should be based on widely-accepted standards and therefore implicitly increasing compatibility with standard based security policies.
F7	Exchange of data between organizations is based on agreements following GDPR	The design shall take GDPR into account wherever applicable (e.g. in the design of the consent process).
F8	Common mechanism of eID currently built (eIDAS)	The design of the solution shall be based on eIDAS, which is expected to be the de-facto standard among EU Member States.
F9	Cloud services, compatible with medical data exchange legislation	KONFIDO will be able to use cloud infrastructure being compatible with the respective legislation.
F10	Credible network services available	Facilitate the engagement in high mobility scenarios.

### Outcome 3: Recommendations for the technical design

Based on the outcomes from all methodological pillars (Fig. 1), we concluded with a list of recommendations for the design phase of the KONFIDO toolkit. Notably, not all the produced recommendations concern technical aspects that can be overcome in the context of KONFIDO. Some of them are quite generic, exceeding the KONFIDO scope. Nevertheless, since these can be useful for the designers of cybersecurity tools in the health domain, we cite below the full list of recommendations:

1. Strive for high adherence to standards as this reinforces end-users’ trust in HIT.
2. Leverage existing technical frameworks of the domain (in the European context, e.g. OpenNCP and eIDAS), but also follow a flexible design to address technical dependencies to the extent possible.
3. Implement state-of-the-art cybersecurity technologies and measures, while ensuring sustainability of the technical solutions.
4. Consider how and where consent is registered as well as accessed by patients and HCPs.
5. Adopt a clear and comprehensive data handling scheme, in order to facilitate its understanding (for both patients and HCPs).
6. Usability should be a first-class priority in cybersecurity technical developments, given that this constitutes a key acceptance factor for the end-users.
7. Implementation details should target the three pilot countries as there are too many open issues to plan and conclude in the development of an EU-wide robust technical solution. A prototype toolkit targeting the three pilot countries can be used as an example for the future development of an EU-wide solution.
8. Carefully take into account the diversity of organizational and information workflows applied in healthcare organizations, and adapt the technical design accordingly.
9. Comply with all applicable laws and regulations in the involved regions and countries, but also with EU regulations related to HIT. At the same time, be adaptable to prominent changes regarding legal issues and take into account that legislation is not aligned among EU Member States.
10. The lack of budget to address security aspects by healthcare organizations dictates that new cybersecurity technologies shall be cost-effective, contributing to practical solutions.

## Discussion

### Principal results

The current study provided a comprehensive set of user requirements and a set of barriers and facilitators for HIT acceptance associated with the design of secure and interoperable HIT, concluding with recommendations for the technical design phase of cybersecurity solutions focusing on health data exchange and the KONFIDO toolkit in particular.

According to the gap analysis, full adherence with information security standards is currently not universally met. In view of the rapid pace of cybersecurity technologies, sustainability plans shall be defined for adapting existing/evolving frameworks to the state-of-the-art. Overall, lack of integration in a holistic security approach was clearly identified. For each user scenario, a comprehensive workflow has been defined, highlighting challenges and open issues for their application in our pilot sites. The threat analysis resulted in a set of 30, high-level user goals in total, which were documented in detail, while links among our information sources and assets, threats and goals were identified as part of a meta-analysis. The survey and the Workshop with key stakeholders validated the above-mentioned outcomes. Indicative barriers of HIT acceptance include lack of awareness regarding HIT risks and legislations, lack of a security-oriented culture as well as usability constraints, while important facilitators concern the adoption of standards and the efforts to establish a common legislation framework across EU. To this end, GDPR is a significant step forward which will certainly affect the management of patient data and the design of HIT systems. However, its detailed analysis exceeds the scope of our user requirements engineering methodology and, therefore, GDPR is not further elaborated in this paper.

The overall outcomes obtained from the presented user requirements engineering methodology were consolidated as recommendations for the design of cybersecurity solutions. Despite the fact that some of these recommendations do not concern technical aspects that can be overcome in the context of KONFIDO, we stress their importance, as they can provide significant insights for the design and development of cybersecurity solutions in the healthcare domain at large.

### Limitations

As our study relies on multiple methodological steps, various limitations per step can be identified. In particular, the gap analysis study entails the subjectivity in both the obtained responses and the interpretation of the analysis subjects. As a mitigation action, we extensively discussed and tried to clarify cases of vague/unclear input across the respective WGs. When necessary, we contacted the producers of the analysis subjects (e.g. consortia of the considered projects) for clarifications. The employed gap analysis framework (template) did not specifically address cross-border data exchange, storage and management, which is the main objective of our project. In addition, while relying on ISO standards and having an adequate level of detail concerning information security, the employed gap analysis template might not cover all possible conditions. Nevertheless, we believe that potential missing aspects will be identified and addressed as the technical development evolves.

The user scenarios were driven by the current setting of the KONFIDO pilot sites. Given the project setup, the pilot studies for assessing the KONFIDO toolkit will be conducted in three European countries. Thus, it is possible that our analysis missed cybersecurity-related aspects that are applicable in other European countries. In order to overcome this limitation, the conducted end-user survey targeted a broad audience, aiming to obtain input from the widest possible spectrum of stakeholders composing the European eHealth ecosystem.

Overall, as the study of other HIT ecosystems (e.g. the case of exchanging health data among different hospitals in US) is out of the current work’s scope, the European focus of the study can be considered as a limitation per se. Nevertheless, the heterogeneity which is met across the different national healthcare systems in Europe constitutes a unique characteristic that is worth investigating. Our study outcomes could also be generalized and exploited in the context of exchanging data in other contexts, e.g. with other countries outside EU. For example, the Trillium-II project [38], which focuses on EU-US cooperation and particularly on exchanging patient summary data, could find our outcomes useful both regarding barriers, facilitators and end-user goals, as well as our technical advances. Raising awareness about cybersecurity for health data exchange requires intensive synergies, in order to build the necessary cybersecurity-oriented culture and address the respective barriers that were identified in our study.

### Comparison with prior work

To the best of our knowledge, this is the first systematic study presenting and applying a comprehensive, user requirements engineering methodology for the design of secure and interoperable HIT. Our methodology included a broad range of activities, starting from a gap analysis study which reviewed a wide range of relevant projects/initiatives, technological artifacts as well as end-user organizations’ policies and national cybersecurity strategies. User scenarios have been defined and analyzed in detail, focusing on three pilot sites and cross-border health data exchange. The respective user requirements elicitation phase containing a threat analysis of the business processes entailed in the user scenarios, defined assets, threats and, ultimately, high-level user goals. Finally, an end-user survey and a Workshop with the participation of diverse stakeholders validated the obtained outcomes of the previous steps and identified key barriers and facilitators for HIT adoption linked with cybersecurity. Overall, the presented methodology is aligned with best practices [39] and established methods in the domain of requirements engineering for digital health, with respect to requirements elicitation and validation [40], as well as security requirements identification [41].

## Conclusion

This study enabled us to define a comprehensive set of user requirements, a set of barriers and facilitators for HIT acceptance and, ultimately, a set of recommendations for designing a toolkit for secure and interoperable health data exchange in Europe. We argue that our results provide important insights to the domain, while our methodological framework constitutes a paradigm that can be reused for investigating other kinds of cybersecurity-related risks in the health sector. Equally important, the identified barriers and facilitators for HIT acceptance may constitute a useful guide for HIT stakeholders in reinforcing the adoption of their solutions by the targeted end-users (i.e. HCPs and patients/citizens).

## Additional files

Additional file 1: The gap analysis template. (XLSX 36 kb)

Additional file 2: Survey structure for HIT Stakeholders. (XLSX 15 kb)

Additional file 3: Survey structure for Patients-Citizens. (XLSX 14 kb)

## Abbreviations

BP: Business Process
EHR: Electronic Health Record
eID: electronic IDentification
eIDAS: electronic IDentification, Authentication and trust Services
ENISA: European Union Agency for Network and Information Security
epSOS: European Partners – Smart Open Services
EU: European Union
GDPR: General Data Protection Regulation
HIT: Health Information Technologies
HCP: Healthcare Provider
IEC: International Electrotechnical Commission
ISO: International Organization for Standardization
ISO/IEC 27k: The ISO/IEC 27000 suite of standards
IT: Information Technology
JASeHN: Joint Action to Support the eHealth Network
mHealth: Mobile Health
NCP: National Contact Point
OpenNCP: Open-source and reference version of the NCP software
p-PUF: photonic Physical Unclonable Functions
SIEM: Security Information and Event Management
SQuaRE: Systems and software Quality Requirements and Evaluation
STORK: Secure idenTity acrOss boRders linked
STRIDE: Spoofing – Tampering – Repudiation – Information disclosure - Denial of service – Elevation of privilege
WG: Working Group
